# Evolution of the experience with the semiconstrained distal radioulnar joint arthroplasty: A single center review of medium-term outcomes and complications

**DOI:** 10.1016/j.jhsg.2026.101117

**Published:** 2026-09-03

**Authors:** Martina Greminger, Laima Bandzaite, Inga Swantje Besmens, Gabriella Fischer, Maurizio Calcagni

**Affiliations:** ∗ Department of Plastic- and Hand Surgery, University Hospital Zurich, Zurich, Switzerland; † Institute for Biomechanics, ETH Zurich, Zurich, Switzerland

**Keywords:** Aptis DRUJ arthroplasty, Aptis prosthesis, Distal radioulnar joint arthroplasty, DRUJ arthroplasty, Scheker prosthesis, Semiconstrained DRUJ prosthesis

## Abstract

**Purpose:**

Replacement of the distal radioulnar joint (DRUJ) using a semiconstrained implant arthroplasty (Aptis) has demonstrated the capacity to restore function. The aim of this study was to investigate medium-term outcomes and navigate the effect of increasing experience with this implant, and compare the results with other follow-up studies.

**Methods:**

We retrospectively investigated 36 DRUJ implant arthroplasties in 34 patients operated on between 2010 and 2021 for complications and revision surgeries. The average follow-up time was 4.4 years. In 15 patients with available data and surviving implant over time, we compared pre- and postoperative pain level and total active motion, as well as weight-bearing ability.

**Results:**

Overall pain reduction was significant. Active range of motion and weight bearing ability was stable over time. We observed no infection. Revision surgery was necessary in 27% of cases, and the overall survival rate of the implant was 91% with a mean follow-up of 53 months.

**Conclusion:**

We conclude that implant arthroplasty of the DRUJ reduces pain and preserves function. An extremely precise surgical technique is mandatory to avoid complications.

**Type of study/level of evidence:**

Prognosis/IIb.

The sigmoid notch of the radius and the ulnar head form the articular surfaces of the distal radioulnar joint (DRUJ). The DRUJ allows rotation of the forearm along with the proximal radioulnar joint and the interosseous membrane. It is furthermore essential for the transmission of forces across the forearm.^1^ Different treatment options have been proposed to treat symptomatic DRUJ conditions. The majority consist of partially^2^ or totally^3^ removing the ulnar head or fusion of the DRUJ combined with proximal ulna diaphysis interruption.^4^ The DRUJ may remain painful after these procedures due to ulnar stump instability,^5^ and load-bearing ability may remain restricted.^6^ Another option is replacement of the ulnar head.^7^^,^^8^ The success of these arthroplasties depends on the integrity of the primary stabilizing triangular fibrocarilage complex or equivalent ligament reconstruction. The semiconstrained DRUJ prosthesis (Aptis Medical) described by Scheker et al^9^ is a total joint arthroplasty that replaces all of the components of the DRUJ and provides stability by replacing the triangular fibrocartilage complex function^10^, ^11^, ^12^ and can be used for symptomatic DRUJ arthritis due to trauma, degenerative or inflammatory disease. This cobalt-chrome implant is a ball-in-socket system on the radius combined with a stem within the ulna diaphysis. Previous outcome studies published in the recent years showed variable complication rates ranging between 8% and 33% (Table)^13^, ^14^, ^15^, ^16^, ^17^, ^18^ and good functional recovery.^13^^,^^14^ The aim of this study was to evaluate the subjective, clinical, and radiographic outcomes in patients who underwent surgery with the Aptis semiconstrained DRUJ endoprosthesis between 2010 and 2021. Special attention was given to complications, and the results were compared to previously published outcomes.^13^, ^14^, ^15^, ^16^, ^17^, ^18^, ^19^, ^20^

**Table:** Summary of Previous Studies Reporting Outcomes of the Aptis Implant

Reference/Year of Publication	Implants, n	Implants With Complications, n	Complication Rate Requiring Surgery	Implant Explantation/Replacement	Survival Rate	Average Follow-Up	Type of Complications	Patients With Previous Surgeries	Special Cohort
Rampazzo et al^13^ (2015)	46	15 (requiring surgery)	33%	2	96%	61 mo	ECU tendonitis, infection, periprosthetic fracture, ectopic bone formation, radial plate malposition, lunate implant impingement	80%	Only patients under age 40 y
Bellevue et al^14^ (2017)	52	19 (requiring surgery)	29%	5	90%	1.6 y	Periprosthetic fracture, infection, aseptic loosening, implant component failures, screw loosening, neuroma, finger stiffness, heterotopic ossification	75%	
Kachooei et al^15^ (2014)	14	2 (requiring surgery)	14%	0	100%	60 mo	Screw protrusion	100%	
Warlop et al^16^ (2021)	42	22 (overall); 12 (requiring surgery)	24%	3	92%	46 mo	Tendon irritation/subluxation, nerve irritation, hematoma, CRPS, impingement, VISI, aseptic loosening	80%	
Jawahier et al^17^ (2023)	50	6 (overall); 4 (requiring surgery)	8%	0	100%	29 mo	ECU tendonitis, neuroma, synovitis, ulnar nerve dysfunction	40%	
DeGeorge et al^18^ (2017)	50	22 (overall); 8 (requiring surgery)	16%	2	96%	36 mo	Extensor tendinopahthy, screw protrusion, periprosthetic fractures	92%	

## Materials and Methods

Between 2010 and 2021, 36 DRUJ implant arthroplasties using the Aptis system were performed at our institution on 34 patients (13 females, 21 males) with symptomatic DRUJ conditions. The primary indications for surgery were painful arthritis and/or DRUJ instability. The entire cohort was assessed for complications and revision surgeries. Data were collected retrospectively from patient records and case report forms. The follow-up period ranged from 3 months to 13 years, with an average of 53 months (SD = 40 months). Three patients were lost to follow-up and excluded from the study. Hence, a total of 33 implants were included in the overview of complications.

Fifteen patients with available pre- and postoperative values and surviving implant at the follow-up point were included in the statistical analysis to compare pre- and postoperative pain level and range of motion. The follow-up duration for this subgroup was 76.6 months (SD = 35.25 months).

For subjective assessments, we used the Visual Analog Scale (VAS) for pain ranging from 0 to 10 in severity. Active range of motion (pronosupination) was measured using a goniometer while grip strength was assessed with a JAMAR Hydraulic Hand Dynamometer (Performance Health). Information about concomitant procedures and subsequent revision surgeries was collected from all patient records.

Ethical approval was obtained from the regional ethics committee (Kantonale Ethikkommission Kanton Zürich, BASEC-Nr. 2023-00318). All patients provided written informed consent.

### Surgical technique

The standard procedure for implantation of the semiconstrained DRUJ prosthesis has been previously described.^9^ We adhered to this standard technique in all cases. All arthroplasty procedures were performed by three senior surgeons.^21^ Implant sizes were templated preoperatively by the manufacturer. Postoperatively, all patients wore a removable short arm splint for 6 weeks and began active exercises 2 weeks after surgery.

### Statistical analysis

Descriptive statistics including median and interquartile range (IQR) as well as mean and standard deviation (SD) were calculated for all continuous variables. To compare preoperative and postoperative measurements, we assessed the normality of the data using the Shapiro-Wilk test and employed a Wilcoxon Test. The level of significance was set at *P* ≤ .05.

## Results

**Figure 1:**
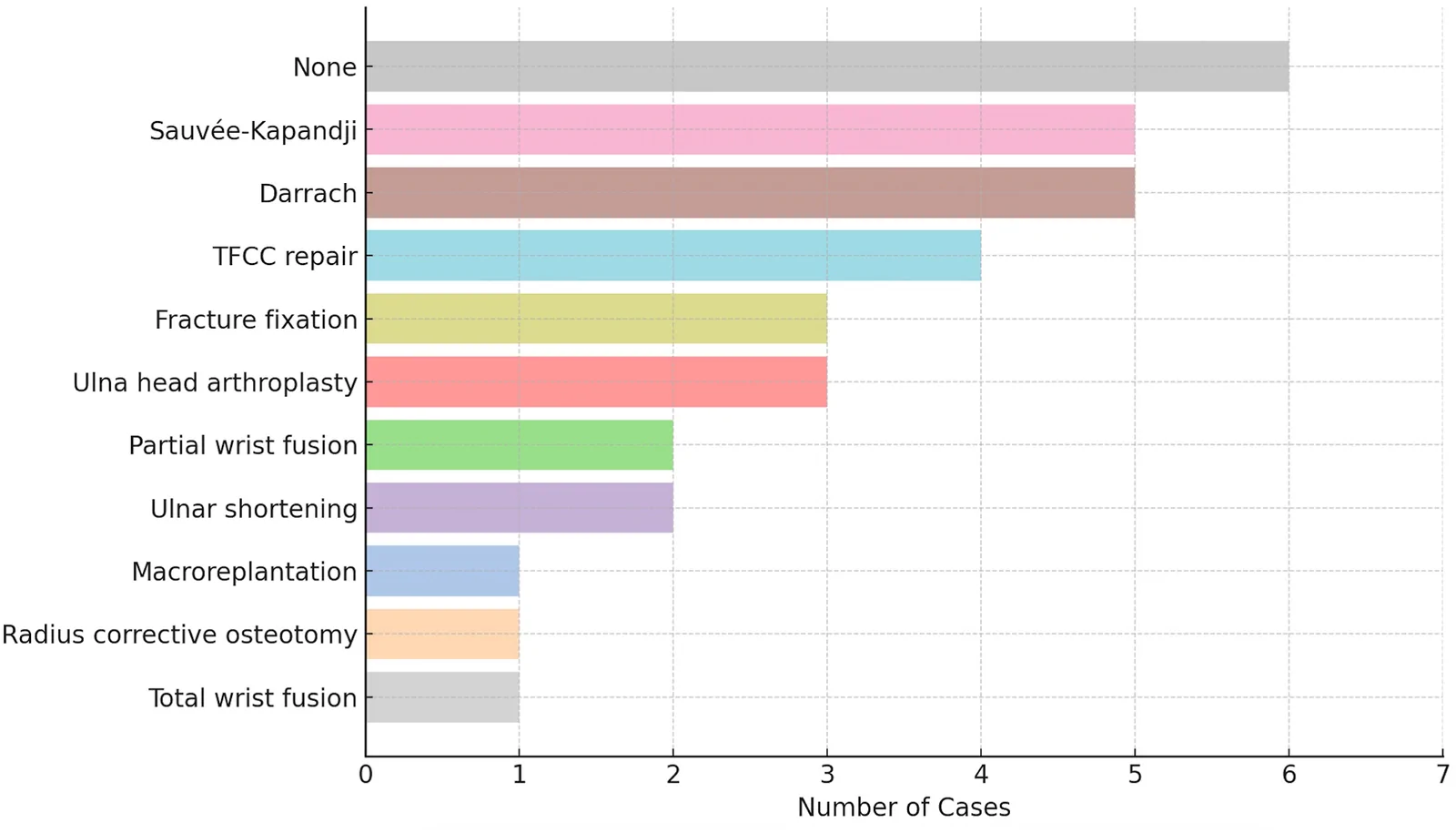
We collected details about concomitant procedures from all patient records. Only five out of 31 patients of our study group did not have a previous operation. All other cases had a least one previous surgery. The most frequent previous surgeries in our cohort include the Darrach and Sauvée-Kapandji procedure as well as TFCC repair. TFCC, triangular fibrocartilage complex.

The average age at the time of surgery was 46 years (SD = 11 years). Sixteen implants were inserted in the right forearm and 17 in the left. Of the 31 patients (33 implants) included in our cohort, only five patients (six implants) had no previous wrist or DRUJ surgery. The remaining 26 patients (27 implants) had undergone at least one prior operation (Fig. 1), four of the cases even multiple previous surgeries.

### Patient-reported outcomes

**Figure 2:**
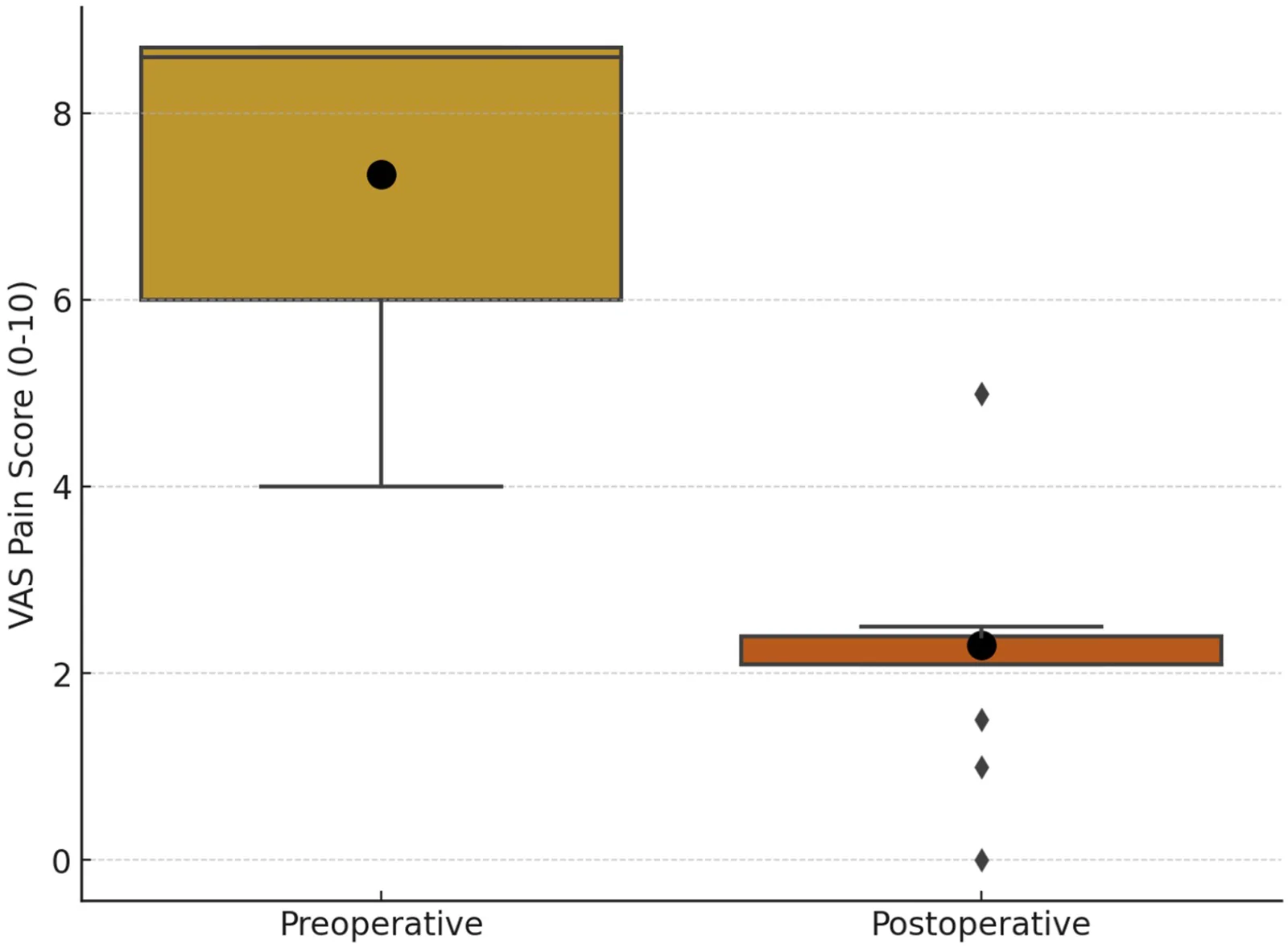
We evaluated pain at rest on a VAS before surgery and at the follow-up examination. On the VAS, pain at rest improved significantly (*P* < .001) from a preoperative mean of 7.4 (SD = 1.7) to 2.3 (SD = 1.3)

Pain at rest, measured using the VAS score, improved significantly (*P* < .001) from a preoperative median of 7.35 (IQR = 6.0−8.7) to a postoperative median of 2.31 (IQR = 2.1−2.4) (Fig. 2).

### Clinical outcomes

**Figure 3:**
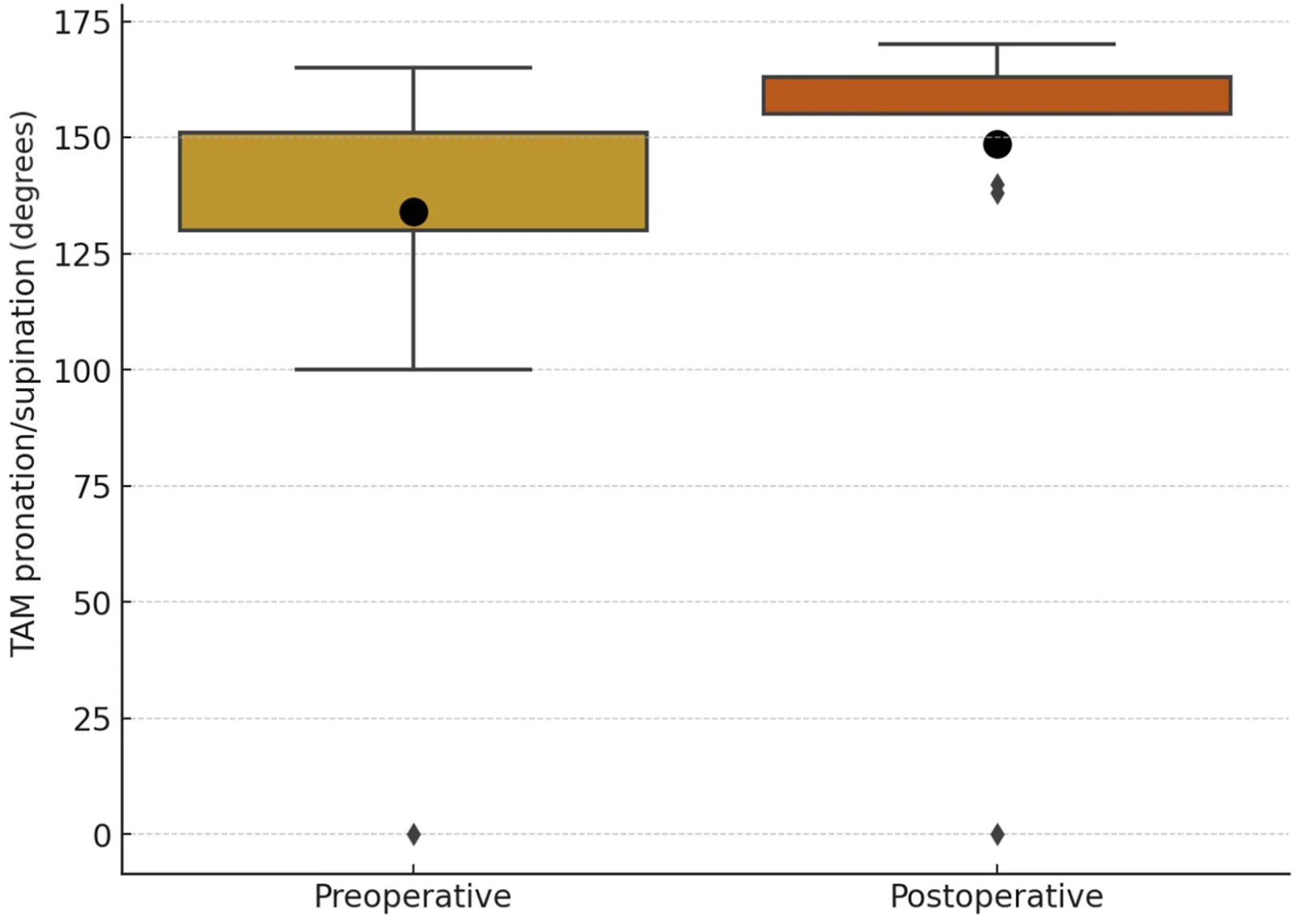
We evaluated wrist motion before surgery and at the follow-up examination. Total active motion in pronation/supination improved significantly from preoperative 134° (SD = 41°) to postoperative 149° (SD = 42°) at the follow-up time point.

Total active motion in pronosupination improved significantly from preoperative median 151° (IQR = 130°−151°) to postoperative median of 163° (IQR = 155°−163°) at the follow-up time point (*P* = .008) (Fig. 3).

**Figure 4:**
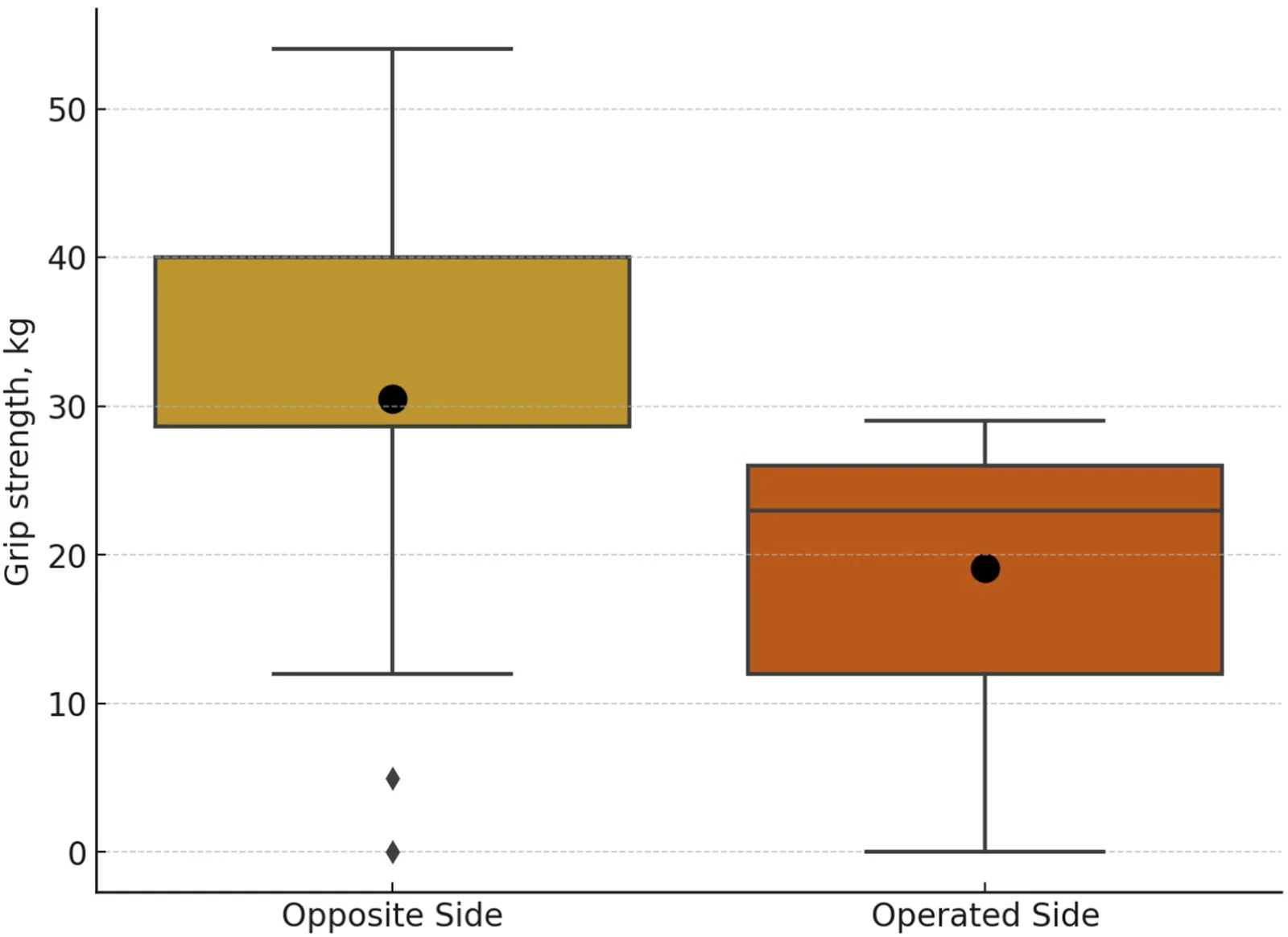
Grip strength was evaluated with a Jamar dynamometer at the follow-up examination and compared to the contralateral side. Jamar grip strength of the operated wrist was on average 19 kg (SD = 8.5 kg) and was 62% compared to the contralateral side (mean, 30.5 kg; SD = 14 kg) at the follow-up time point.

The median Jamar grip strength of the operated wrist was 23 kg (IQR = 12−26 kg), which was 80% of the contralateral side’s strength (median, 28.6 kg; IQR = 28.6−40 kg), *P* < .0001 (Fig. 4).

### Complications

Among the 33 implants placed in patients between 2010 and 2021, we observed a total of nine complications, yielding an overall complication rate of 27%. Four of these cases were already described in recent investigations in 2016 and 2020. In 2016, three complications out of 10 patients were observed: one patient with ulna stem loosening requiring revision surgery with cementation of the ulnar stem. Additionally, two patients underwent reoperation due to extensor tendon irritation and involvement of the superficial branch of the radial nerve.^19^ In 2020, three new complications were identified in the same cohort, in addition to those previously reported on. The patient who had undergone revision for ulna stem loosening with cementation experienced reloosening, necessitating prosthesis removal. Another patient had an allergic reaction to the metal alloy following screw shortening, which had been performed with a diamond burr to address nerve irritation from screw protrusion. This patient also required prosthesis removal. Furthermore, implant revision was necessary in one case of ulnar impaction of the implant due to excessively distal placement. After proximal repositioning with exchange of the implant, the patient experienced significant pain relief and improved hand function. At that point, six complications across four patients were observed (with one patient lost to follow-up), leading to two revision surgeries and two implant removals. This resulted in a complication rate of 40% in an earlier investigation.^20^

Between 2014 and 2021, an additional 24 DRUJ arthroplasties in 22 patients were performed. All of these cases were not included in the previous investigations. In this cohort, six complications were identified in five patients, all of which required revision surgery. This led to a complication rate of 22% in the later cohort. Notably, none of these complications necessitated implant removal or replacement.

**Figure 5:**
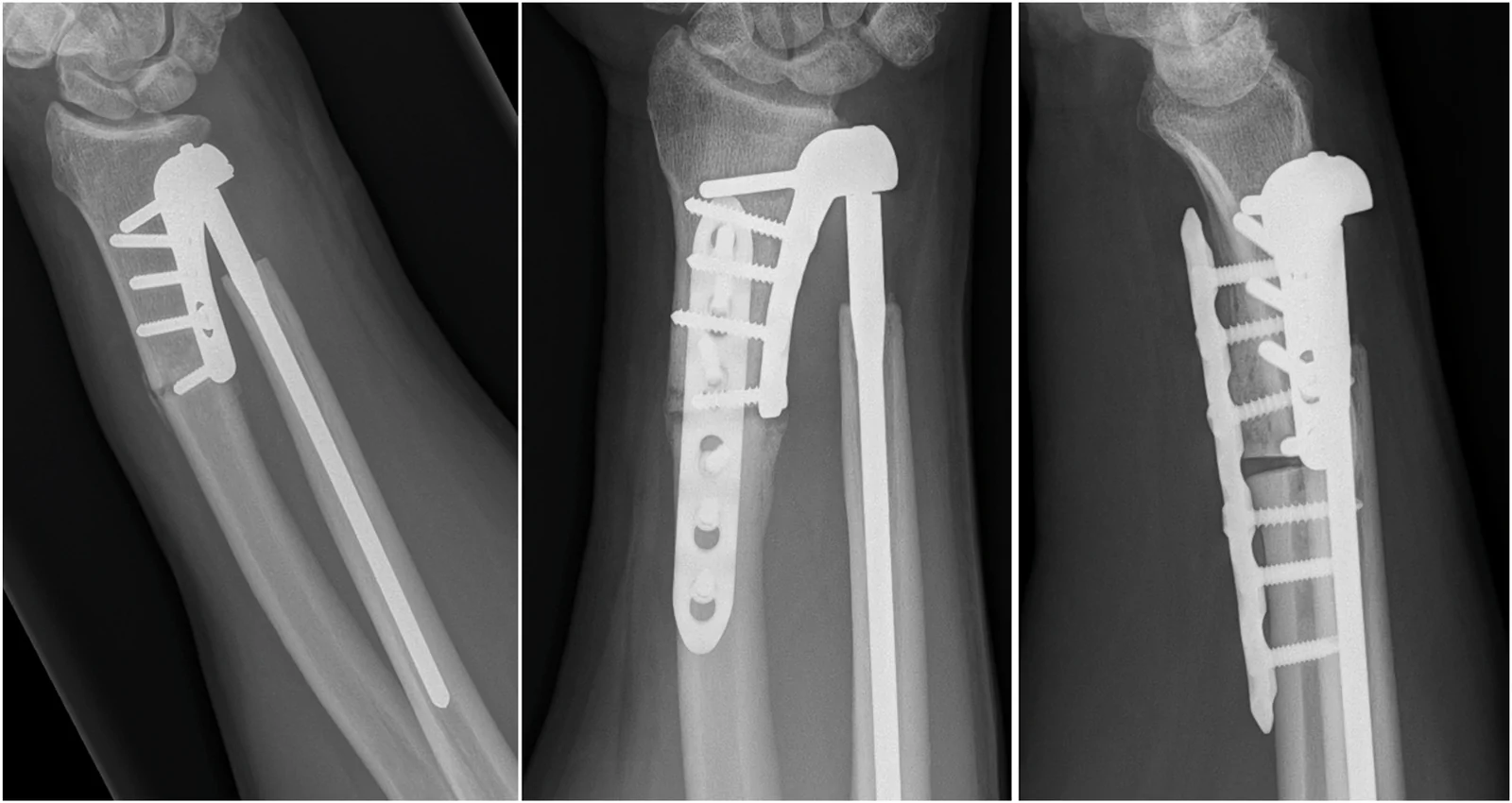
We observed one case with a periprosthetic fracture at the level of the most proximal screw of the radius. This screw is a stress-riser, and therefore, unicortical screw placement is recommended in this position. The initially conservatively treated fracture led to a symptomatic malunion and a corrective osteotomy was necessary.

Two patients in the later cohort sustained periprosthetic fractures, one due to trauma and the other during push-up exercises after DRUJ implant arthroplasty. One of them required surgery for fracture fixation, and the other patient was treated conservatively (Fig. 5). Both fractures healed completely. However, the initially conservatively treated patient developed a malunion with restriction of the pronosupination, so a corrective osteotomy was necessary 1 year later with improvement of rotation to the previous values.

**Figure 6:**
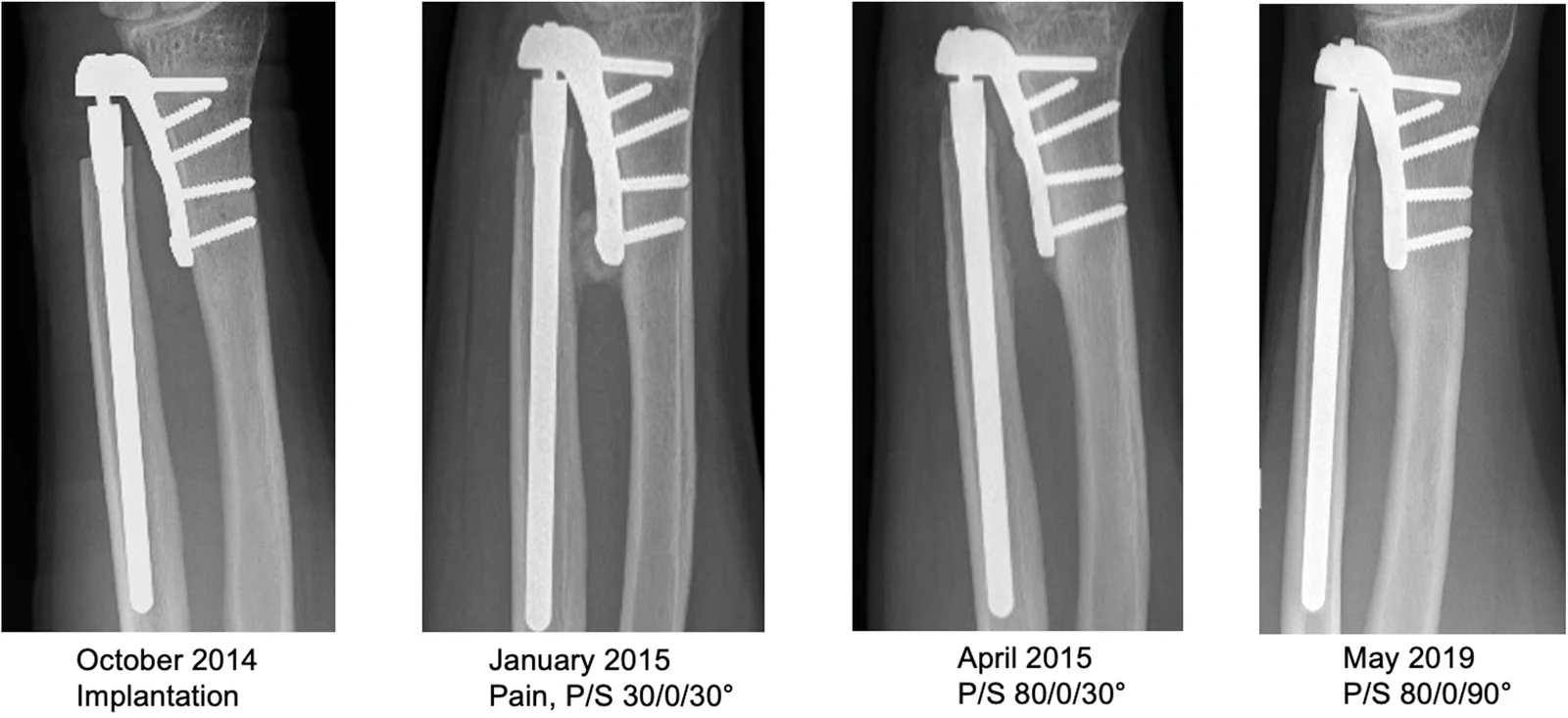
One patient suffered from heterotopic ossifications located at the proximal border of the radius plate, leading to a limitation in forearm rotation. These ossifications occurred only within 6 months after implantation. After excision and interposition of a fascial flap pronation and supination improved from 30/0/30° to 80/0/90°. No recurrence was observed.

Two patients developed heterotopic ossification, presenting with recurrent pain as well as limited range of motion several months postoperatively. Both required excision of the heterotopic ossifications. In one case ossifications formed at the proximal border of the radius plate between the radius and ulna (Fig. 6), limiting forearm rotation. Surgical excision with fascial flap interposition led to an improvement in pronosupination from 30/0/30° to 80/0/90°.

Furthermore, screw loosening was observed in one case, requiring screw exchange. One patient suffered from recalcitrant extensor carpi ulnaris (ECU)-tendonitis unresponsive to conservative measures and a steroid infiltration. This patient ultimately required ECU tendon resection, resulting in complete pain relief with no subjective loss of function.

**Figure 7:**
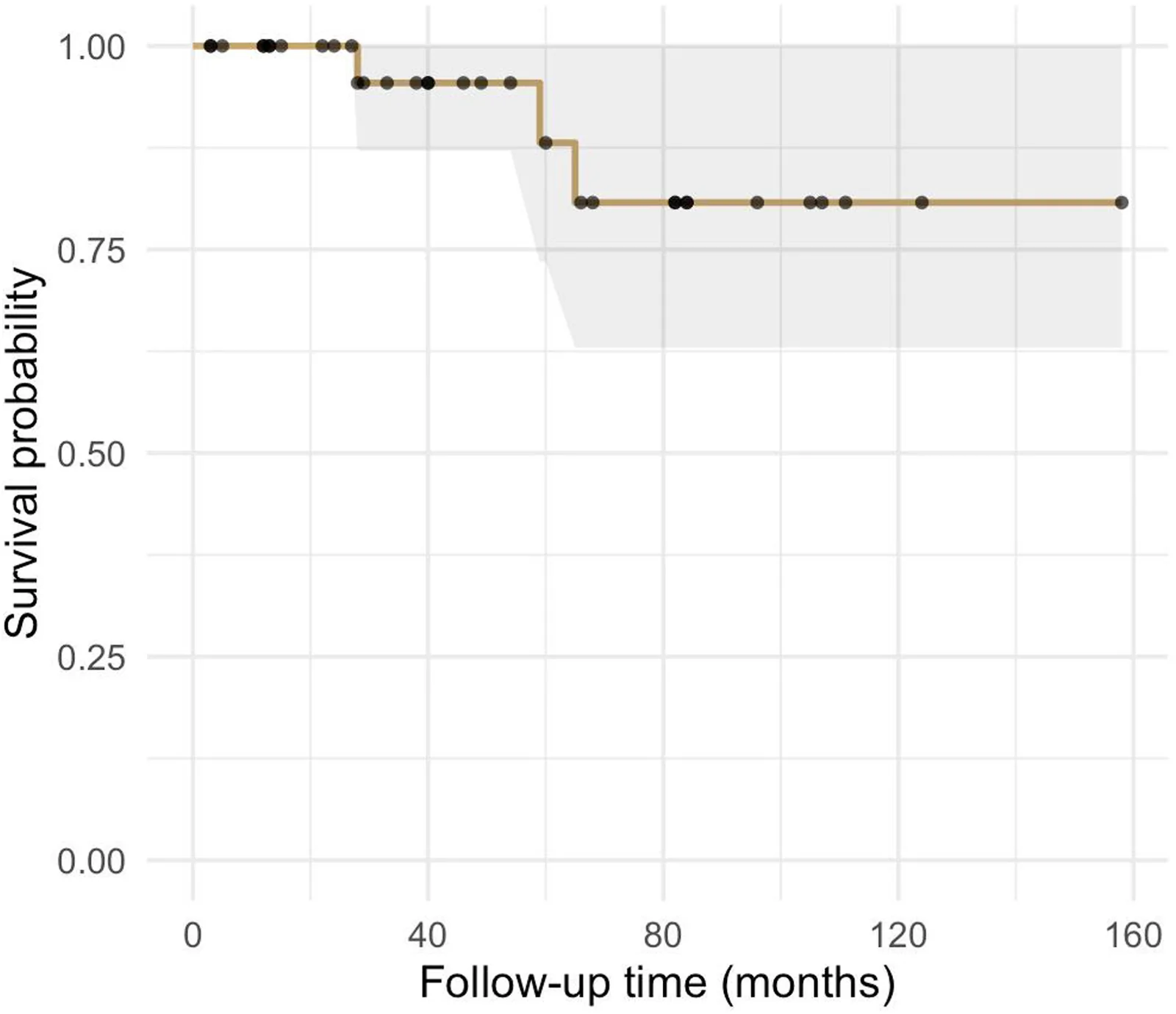
Kaplan-Meier curve of our cohort. Overall survival rate was 91% at a mean follow-up of 53 months. Two implants were removed at 28 months and 59 months, respectively, whereas one implant was exchanged at 65 months.

Overall, two implants were removed at 28 months and 59 months respectively, whereas one implant was exchanged at 65 months. This is schematically illustrated on the Kaplan-Meier curve (Fig. 7) and corresponds to an overall survival rate of 91% at a mean follow-up of 53 months.

## Discussion

### Implant survival

We observed an overall 4.4-year survival rate of the implant of 91% in our cohort, and a reoperation was necessary in nine out of 33 patients (27%). Compared with the results published in 2016 and 2020,^19^^,^^20^ we observed a lower complication rate in the current investigation. This indicates an evolution of the experience with this technically demanding procedure over time.

In the literature, comparable 5-year survival rates and complication rates are documented across different investigations (Table). Most of the implants were used in secondary surgeries and multioperated wrists. Only the cohort of Jawahier et al^17^ differs from the other investigations. In this cohort, the majority of cases had no prior surgeries (60%). The outcomes reported in this study demonstrate above-average results, with a complication rate of 8% and an implant survival rate of 100%. However, the average follow-up period at 29 months is shorter compared to other studies. We share the conclusion of the authors that more research is necessary to assess the value of the implant as primary procedure.

### Complications

#### Technical complications

In 2016, two patients with nerve and tendon irritation problems due to screw protrusion were observed.^19^ These problems can be prevented with increasing experience with the implant and refinement of the surgical technique. Kachooei et al^15^ similarly reported on two implants with radial screw overlength, both underwent secondary surgeries with debridement of the screw tip over the radius. DeGeorge et al^18^ observed that soft tissue and nerve-related complications were most common in their cohort. The authors reported on extensor tendinopathy in five out of 50 arthroplasties and attributed it to a prominent screw in three of these cases. Prominent screws affected the tendons of the first and second dorsal compartment. The prominent screws were removed and replaced. The authors recommended to measure and confirm screw length with fluoroscopy to prevent this complication. They also stressed on the importance of proper positioning of the radial plate: the volar edge of the plate should be aligned with the volar cortex of the radius in the lateral view. Malposition of the radial plate was shown to cause synovitis in the fourth through sixth dorsal compartments.

In our current cohort, we observed one patient with recalcitrant ECU-tendonitis, resulting in ECU tendon resection. We used a retinacular flap to cover the prosthesis in all primary surgeries. Rampazzo et al^13^ reported on nine cases of ECU-tendonitis, which was treated with an ECU sheath release and coverage with a dermal-fat graft or a local adipofascial flap. ECU tendonitis was the main complication in their group of 46 arthroplasties, which occurred in 19% of cases. The authors encountered ECU tendonitis only when the adipofascial flap had not been used primarily. They concluded that the tendonitis may have been caused by direct contact with the implant and may be avoided by dissecting a local adipofascial flap at the beginning of the surgery to cover the implant. DeGeorge et al^18^ reported on three cases requiring extensor tenosynovectomy. The authors primarily created an ulnarly based extensor retinacular flap from the second to sixth dorsal compartments upon approach to the DRUJ as preventative measure. However, creation of this flap was not always feasible, particularly in patients with multiple previous operations. In these patients, the authors recommended placement of a tensor fascia lata allograft or acellular dermal matrix construct to protect the dorsal compartments from interacting with the implant.

#### Nontechnical complications

In our latest cohort, we observed two cases of periprosthetic fractures. This complication is hard to avoid; therefore, patient information about the risk and maximal load capacity of the implant is crucial. Bellevue et al^14^ also reported on periprosthetic fractures. Two of the observed cases with periprosthetic fractures occurred at the level of the most proximal screw in the radius. The authors concluded that the most proximal screw is a stress-riser and recommended placement of a unicortical screw in this position to decrease the risk of periprosthetic fracture. In our patient cohort one periprosthetic fracture occurred similarly at the level of the most proximal radius screw, while the other one occurred in the midthird of the radius diaphysis while doing push-ups. This patient had a previous trauma with a midforearm fracture requiring multiple surgeries, most likely resulting in a weakness in this part of the radius.

### Outcome measurements

Clinical and subjective outcomes in our recent cohort indicate a significant reduction of pain with an improvement of pronosupination postoperatively. Previous studies also reported postoperative pain relief,^13^^,^^19^^,^^20^ and variable effects on the mobility of the wrist after DRUJ implant surgery. Recurrent pain or severely limited range of motion after the operation was occasionally associated with complications. In these cases, pain relief or adequate wrist range of motion could be restored by revision surgery (excision of the heterotopic ossifications [n = 2], resection of the ECU tendon [n = 1]).

As loads during gripping and lifting are transmitted through the DRUJ, it was assumed that the reduced pain level enables for a higher application of grip force. In our cohort Jamar grip force was not improved after DRUJ implant surgery, despite improvement in pain scores. However, a limitation of our study is that we assessed pain level only at rest, not during grip. As discussed by Axelsson et al,^22^ diverging results in the literature do not allow to draw any conclusions whether an improvement, no change or even a reduction in grip force occurs after DRUJ implant surgery. The correlation between Jamar grip strength measurement and patient-reported outcome was weak.

Another limitation of our study is that clinical outcome analyses rely on a smaller subgroup of only part of the cohort, driven by limited data availability for either preoperative or postoperative values in range of motion or grip strength. This bears a potential risk of survivorship and selection bias.

Overall, we observed similar complications as described by other investigations. Some of these can possibly be avoid by precision of the surgical technique and advanced experience with this implant. The semiconstrained DRUJ prosthesis is often used in patients who have already undergone repetitive DRUJ surgery. Unfortunately, our cohort was too small to assess if patients without previous surgeries have better outcomes. Further investigations with larger cohorts could evaluate this interesting question.

## Conflicts of Interest

No benefits in any form have been received or will be received related directly to this article.
